# Self-Flushing in EDM Drilling of Ti6Al4V Using Rotating Shaped Electrodes

**DOI:** 10.3390/ma12060989

**Published:** 2019-03-26

**Authors:** Manu Goiogana, Ahmed Elkaseer

**Affiliations:** 1 IK4-Tekniker, Advanced Manufacturing Technologies Unit, Iñaki Goenaga 5, 20600 Eibar, Spain; mgoiogana@gmail.com; 2 Karlsruhe Institute of Technology (KIT), Institute for Automation and Applied Informatics (IAI), Karlsruhe 76344, Germany; 3 Port Said University, Faculty of Engineering, Department of Production Engineering and Mechanical design, Port Fuad 42526, Egypt

**Keywords:** EDM, Ti6Al4V, rotating shaped electrode, self-flushing, debris egress, MRR, tool wear, hole taper angle

## Abstract

This article reports an experimental investigation of the efficacy of self-flushing in the Electrical Discharge Machining (EDM) process in terms of tool wear rate (TWR), hole taper angle and material removal rate (MRR). In addition to a plain cylindrical shape, electrodes of different cross sections (slotted cylindrical, sharp-cornered triangular, round-cornered triangular, sharp-cornered square, round-cornered square, sharp-cornered hexagonal and round-cornered hexagonal) were designed as a means of inducing debris egress and then fabricated in graphite. EDM drilling trials using the rotating shaped electrodes were carried out on a Ti6Al4V workpiece. The results revealed that, although a low TWR and minimum hole taper angle were achieved using a plain cylindrical electrode, the usage of rotating shaped electrodes provided self-flushing of the dielectric fluid during the EDM process, which led to an improvement in MRR compared to that achieved with a plain cylindrical electrode. Besides, in general, the electrodes with rounded corners are associated with a lower TWR, a lower hole taper angle and a higher MRR when compared to the electrodes with sharp corners. Considering these results, it was concluded that different process attributes, i.e., TWR, hole taper angle and MRR, are all greatly affected by the electrode shape, and thus, the proper selection of the electrode shape is a precondition to attain a specific response from the EDM process.

## 1. Introduction

There is a growing tendency to exploit advanced materials in a wide range of applications, e.g., aerospace, marine and power engines. This is due to their superior attributes in terms of high strength/weight ratio, wear and shock resistance, and thermal and electrical properties that enable these materials to fulfil critical requirements under severe working conditions [1,2]. The titanium-based superalloy Ti6Al4V is a good example of such a material, one of the so-called difficult-to-cut materials; however, its unique mechanical properties preclude the economical processing of this superalloy using conventional machining processes [3,4,5]. 

Electrical Discharge Machining (EDM) erodes a workpiece material by applying electric sparks regardless of the material’s mechanical properties, and it has shown a good potential in producing real, 3-D, intricate features in advanced difficult-to-cut materials [5,6,7]. In EDM, the mechanism eroding the workpiece material is a thermoelectric process between electrical, conductive electrodes (the tool and workpiece). This produces discrete electric discharges between the electrodes and generates a high temperature plasma channel, where instantaneous thermal dissipation takes place. The local high temperature melts both the workpiece and tool [8]. Then, the eroded material solidifies in the form of debris (solidified eroded particles), and finally, the flushing of a dielectric fluid evacuates the debris [7]. This process occurs repeatedly, enabling a continuous machining of the workpiece. Thus, EDM is considered an enabling technology to effectively process difficult-to-cut materials such as Ti6Al4V and Inconel alloys [9,10]. Micro-EDM has also shown a high potential in producing complex features with high characteristics at the microscale [2,8] regardless of the mechanical properties of the workpiece materials, e.g., hardness or high-strength materials [2]. 

A range of governing parameters, e.g., voltage between the electrodes, maximum current, interelectrode gap, flushing dielectric, charge frequency and duty cycle, all influence the EDM process [2,3,11]. Nevertheless, among these EDM process parameters, flushing of the interelectrode gap is one of the most significant variables [12,13]. An efficient flushing removes the debris from the gap and, thus, enables the continuity of the eroding process [2] and helps maintain a high-precision EDM process. That is, the ejection of debris from the machining gap is important for a high performance of the EDM process, especially in the case of deep sinking/drilling operations [13,14,15]. Varying some EDM parameters mentioned above, like duty factor and charge frequency, can induce local flushing in the interelectrode gap, but this research work focused on the self-flushing phenomenon provoked by different electrode geometries.

It is worth emphasizing that a strong flushing does not necessarily lead to optimal machining: A very high dielectric pressure could continually sweep away the plasma channel leading to a low material removal rate (MRR), and for this reason, flushing has to be implemented in a suitably controlled manner [16]. The presence of debris contamination in the interelectrode gap has a trade-off effect on the productivity of the EDM process. The presence of an appropriate amount of debris could initiate some “random” discharge sparks, which in turn could lead to a higher MRR. In contrast, in the worst-case scenario, this debris could induce short circuits with the subsequent stoppage of the process that would lower the MRR [17,18]. Therefore, one can argue that appropriate flushing leads to a high EDM performance in terms of maximum MRR, a low tool wear rate and the high-precision EDM of difficult-to-cut alloys. 

In view of the importance of flushing on the performance of the EDM process, this research study examines the effectiveness of self-flushing techniques (in terms of tool wear rate (TWR), hole taper angle and Material Removal Rate (MRR)) using rotating shaped electrodes.

The structure of this paper is as follows. Firstly, the state-of-the-art flushing approaches are reviewed, the proposed approach reported in this paper is located within the context of the existing literature and the hypothesis examined is also given. Then, the experimental work is presented, followed by the analysis and discussion of the obtained results. Next, conclusions are drawn, and finally, possible future perspectives are outlined. 

## 2. State-of-the-Art Flushing Approaches

Different approaches have been applied to develop a flushing process and to maximise the MRR during the EDM process. A nozzle outside the machining zone can direct a jet of dielectric towards the gap between the electrodes [19]. However, this is not an effective technique for large depths of machining because the flushing pressure exerted is insufficient for an effective removal of the debris [17]. 

As for internal flushing, Munz et al. [12] investigated the use of flow rates between 5 L/h and 25 L/h on debris removal and process performance. They found that increasing the dielectric flow rate improved decontamination in the electrode gap, flushing away gas bubbles and debris more rapidly, which improved the process performance in terms of the feed rate. Nonetheless, it was also found that an excessive flushing turned into a negative process behavior and caused a drop in the feed rate.

Flushing introduced through a multi-hole bundled electrode when machining Ti6Al4V was proposed by Gu et al. [20], and it was concluded that the proposed technique allowed for a better EDM performance process compared with conventional EDM using a solid electrode. Similarly, Li et al. [21] also experimentally tested the response of EDM processes to the use of a multi-hole bunched electrode and showed how using this special tool affected flushing and the machining performance in terms of TWR and MRR. It was found that the multi-hole bunched electrode was able to withstand a greater peak current, giving a higher MRR but a greater TWR than a conventional solid electrode with single hole flushing. Nevertheless, the multi-hole inner flushing technique needs a special clamp for each external profile of the shape to be machined.

For the EDM drilling of deep holes, Tanjilul et al. [22] suggested an innovative system for debris removal, combining simultaneously flushing and vacuuming. They achieved improved surface quality and better drilling times using such a system. These researchers also developed a novel computational fluid dynamics (CFD) model to simulate the performance of the proposed debris removal system.

Ultrasonic vibrations have been suggested as a way to improve the flushing of the EDM process, especially for difficult-to-cut materials [23] and high aspect ratio blind holes [24]. This technique has shown promising increases in MRR [13], a higher process efficiency [25] and significant improvements in the obtainable surface quality in EDM finishing operations [26]. Although ultrasonic assisted EDM processes are widely used in industry, further investigation is still needed to help understand the ultrasonic vibration-assisted EDM process, dominant variables, process modelling and optimization, especially its application to deep micro/conventional drilling [27].

Other EDM techniques have included using planetary motion (lateral movement of the tool that is of similar geometric shape to that being machined), with a rotating tool to obtain a more homogenous distribution of the tool wear and to improve productivity [28]. Self-flushing using an electrode planetary motion was implemented to produce blind micro-holes with a high aspect ratio [29]. Nevertheless, using this technique, the removal rates were restricted [17]. 

Ziada and Koshy [17] suggested a novel method inspired by the properties of the Reuleaux Triangle (RT) that enabled flushing to be induced by the rotation and relative movement of the electrodes. This pioneering arrangement allowed the machining of both regular and non-regular shapes with sharp corners. The use of the RT electrodes improved flushing and helped make the MRR independent of machining depth. However, it required a complex tool and trajectory design, and further applied investigation work should be done.

An alternative flushing approach is to periodically retract the tool during the machining process to allow the removal of the debris and adulterated dielectric. Masuzawa and Heuvelman proposed the self-flushing method in which the tool motion could take place in more than one axis. This motion could be controlled in a way that the electrode with these movements acts as a pump, and in this way, the gap was continually regenerated [30]. However, the main limit on this technique was that there was a loss of machining time [17]. They did not use any rotatory movement of the electrode.

Another method is to use a rotating electrode to remove debris. Dwivedi and Choudhury [31] investigated both theoretically and experimentally the machining of high chromium and high carbon AISI D3 steel using rotating cylindrical electrodes. This technique resulted in a higher MRR because of the improved debris clearance and spark quality. These researchers claimed that the use of a rotating tool increased MMR by 41% and reduced surface roughness (Ra) by 12% comparing with the values obtained using stationary electrodes. Nonetheless, these tests were carried out in 5 mm thick metallic flats, and the results might vary in high aspect ratio blind holes.

Pellicer et al. [32] proved that, in micro EDM, it is possible to improve the flushing by shaping the tool/electrodes such that the electrode was capable of cutting features in minimum time. Shaped electrodes, the so-called self-flushing electrodes, could effectively remove accumulated debris from the machining zone and, thus, eliminate the need for flushing during the machining process [33]. Plaza et al. [34] tested the effect of using an electrode of helical shape on electrode wear, MRR and micro-hole quality for EDM of Ti6Al4V. The goal was to improve the efficiency of the removal of debris from deep holes and, thus, to reduce the machining time. The parameters investigated were flute depth and helix angle. With a hole diameter of 800 μm, a reduction in the machining time of 37% was obtained with a helix angle of 45°. Kumar and Singh [33] fabricated a new type of electrode with inclined micro slots cut in a solid cylindrical electrode. The tool was used to drill blind holes into a Ti6Al4V workpiece, and the effects of slot width, angle and tool speed were investigated with the goal of improving EDM performance. It was found that the use of inclined micro slots successfully eliminated any accumulated debris, reducing the likelihood of short-circuiting and arcing. The new design was so successful at eliminating debris that the authors claimed it was self-flushing. 

Nastasi and Koshy [35] have suggested improving rotation-induced debris removal by developing novel electrodes of suitable shapes. Flow fields were simulated using CFD in order to explore the ability of radial and helical slots introduced into rotating cylindrical tools to enhance MRR when drilling blind holes in 6061 aluminum alloy. The model suggested and the experiment verified that the tool with a single radial slot achieved the best flushing, and this design gave an astounding 300% increase in MRR compared to a typical cylinder-shaped electrode rotating at the same speed. This was achieved in holes with an aspect ratio of 3.

**Figure 1:**
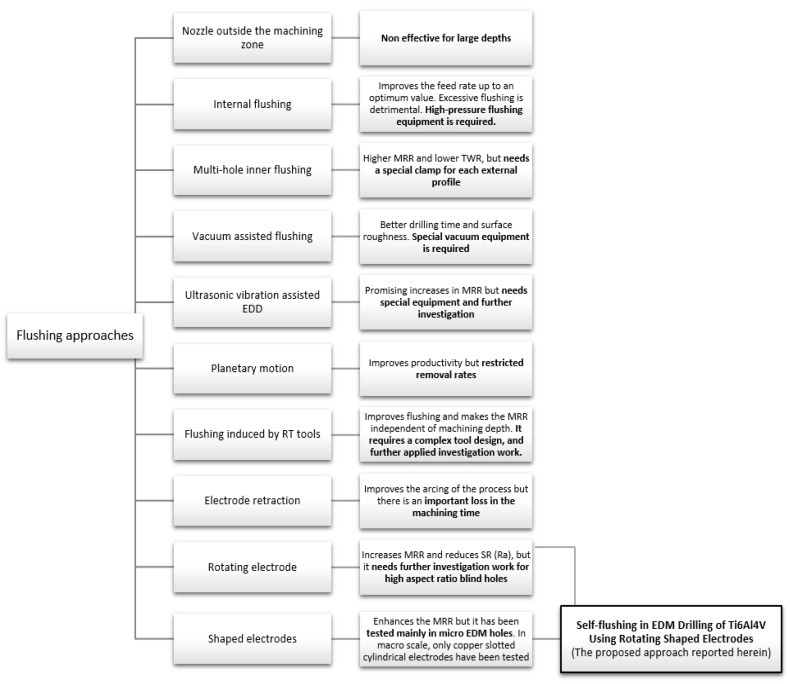
An overview of the state-of-the-art flushing techniques in the Electrical Discharge Machining (EDM) process and the positioning of this research work within the context of the reported literature.

Figure 1 schematically summarizes the state-of-the-art flushing techniques in the EDM process and locates the proposed approach reported herein within the context of the existing literature.

As it is possible to observe in Figure 1, different approaches to flushing have been proposed. Nonetheless, some of them are complex or need special equipment, and others are expensive in terms of the electrode design and fabrication time and effort. Moreover, some of the proposed techniques need further investigative work in order to be implemented and applied in the Electrical Discharge Drilling (EDD) of high aspect ratio blind holes [36]. From the reviewed literature, it is not so difficult to see that the performance of the EDM process is highly related to the debris evacuation process. Accordingly, the hypothesis followed in this research is that using rotating shaped electrodes will induce a self-flushing process. This, in turn, could improve the flushing circulation and quick cleansing of debris from the sparking gap and, thus, contributes to the enhancement of the MRR of the EDD process compared to that obtained applying a plain cylindrical electrode. However, it is not expected to improve the TWR and hole taper angle simultaneously due to the smaller cross-sectional area of the shaped electrode when compared with the plain cylindrical one. In this context, the motivation for this research study was to examine the effectiveness of a self-flushing technique to enhance the machining process, combining the rotation and shaping of the electrodes.

In order to induce self-flushing with rotating shaped electrodes during the EDD tests, the ease of manufacturing the electrodes has been considered when defining the cross-sectional geometry. Further details of the different electrode geometries can be found in Section 3.2.

The performance of the proposed electrode shapes was examined, and their influence on TWR, hole taper angle and MRR was investigated. Due to the discharge conditions used in these tests of EDD in Ti6Al4V (shown in the next section), the results in terms of surface quality and surface integrity were not analyzed. The applied machining conditions were considered roughing conditions, and thus, it was not relevant to observe the surface quality of the workpiece. The aim of this research work was to improve the flushing using different electrode geometries and to identify the optimal conditions (electrode shape) that positively affects the EDM process responses concerning TWR, hole taper angle and MRR.

## 3. Experimental Setup

### 3.1. EDM Process

The tests for this investigation were carried out in an ONA NX3 EDM machine from ONA Electroerosión S.L. (Durango, Spain) which has a positioning resolution of 1 μm. The tests were performed using POCO EDM-3 graphite electrodes (Poco Graphite, Decatur, TX, USA). More details of the geometries of the used electrodes are provided in Section 3.2.

**Figure 2:**
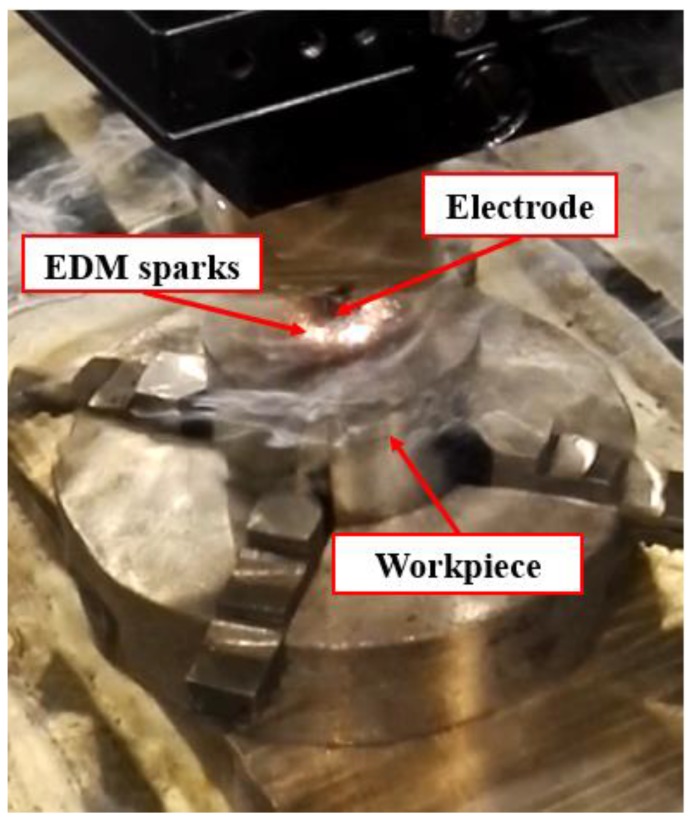
The EDM experimental setup.

The workpiece material used in the experiments was Ti6Al4V titanium alloy. The surface to be machined was ground using a silicon carbide abrasive wheel prior to EDM machining in order to assure the same surface roughness and the same parallelism between the electrode and the workpiece in the different tests (see Figure 2). An X-ray fluorescence system (model S8 Tiger from Bruker, Billerica, MA, USA) was utilized to identify the chemical compositions of the Ti6Al4V workpiece (see Table 1), and the physical properties of the workpiece material are shown in Table 2.

During these tests, 30-mm-deep vertical blind holes with a 14 mm diameter were machined using the graphite electrodes as one of the most common electrode materials for Ti6Al4V [31]. The generic discharge conditions used for these tests are summarized in Table 3 as recommended by the EDM machine manufacturer for EDM drilling with graphite electrode and Ti6Al4V titanium alloy.

### 3.2. Electrode Geometries

To carry out the experimental trials, different electrode geometries were designed and fabricated in order to compare their self-flushing performance during EDM drilling operations. Electrodes with eight different cross sections were used, as shown in Table 4. For inducing self-flushing, it was necessary to select noncircular cross-section profiles. Among all the different possibilities, triangular, square and hexagonal cross sections were chosen considering they were the easiest ones to design and fabricate to make them ready for the EDM tests. The profiles of the cross sections of the electrodes were selected considering the easiest non-cylindrical shapes to machine: triangular, square and hexagonal. In these cases, sharp-cornered and round-cornered cross sections were tested. Apart from that, a slotted cylindrical electrode was analyzed due to the good results obtained with slotted cylindrical electrodes by Nastasi and Koshy [35] during the machining 6061 aluminum alloy. In order to have a reference to compare with, a conventional cylindrical electrode was also examined.

In all cases, the revolving external diameter of the electrode section was 14 mm. In this way, all the electrodes used in these tests had the same effective area. The value of all radii used in round-cornered electrodes was 4 mm, and in the case of slotted cylindrical electrode, the width of the slot was 2 mm and the depth was 5.5 mm. No radius was used in the case of sharp-cornered electrodes. All these electrodes were manufactured in a machining center with a simple milling operation. 

As the main objective was to assess the self-flushing capacity of each different cross section, no external flushing mechanism was used during the tests. This avoids uncertainties associated with external flushing and ensured only the effectiveness of self-flushing was assessed.

**Figure 3:**
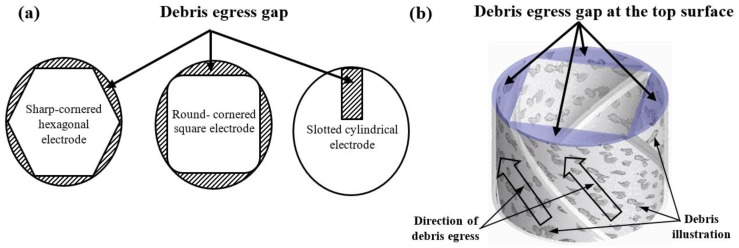
(**a**) The cross-sectional area and the debris egress gap for three different electrode geometries: from the left to the right, the sharp-cornered hexagonal electrode, the round-cornered square electrode and the slotted cylindrical electrode; (**b**) a 3-D representation of the debris egress for the sharp-cornered square electrode.

**Figure 4:**
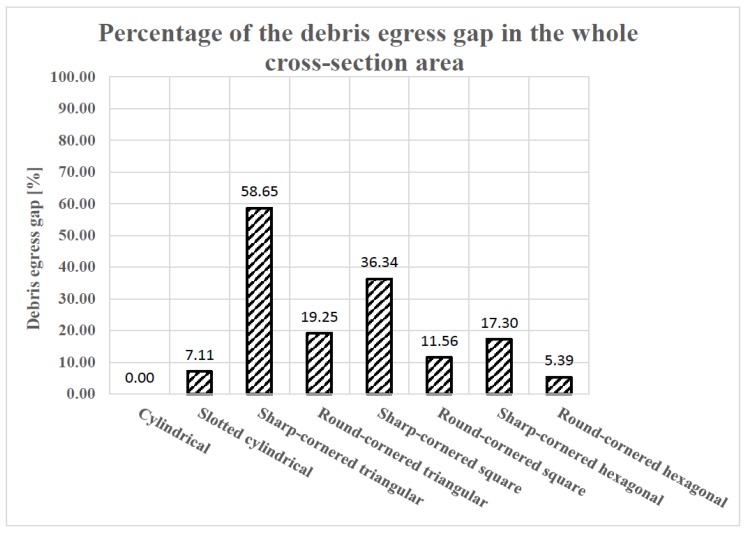
The percentage of debris egress gaps with respect to the whole cross-sectional areas.

The proposed geometry of the eight different electrodes will induce debris egress. In this paper, the difference between the cross-sectional area of the drilled hole and the cross-sectional area of the electrode due to its geometry is termed “debris egress gap”. Figure 3a illustrates examples of the “debris egress gap” for different electrode geometries, and Figure 3b shows a 3-D representation of the debris egress (due to electrode shape and movements) for the sharp-cornered square electrode, which shows how debris is evacuated along the helical paths generated between the moving (rotating and locally retracting) shaped electrode and the machined hole. In Figure 4, the ratio of the debris egress gaps with respect to the whole cross-sectional areas are presented.

### 3.3. Experimental Procedure

During all the tests, the different variables were monitored and recorded, such as axial position and EDM parameters. In addition, in each test, the duration of the operation, removed material and the electrode wear were measured. For that purpose, the EDM machine was connected to a computer via an ethernet cable, and all the parameters were synchronized and recorded in the same Matlab file. After concluding the tests, the collected data were analyzed using the Matlab 2015B package to detect any unusual behaviour during the EDM process, to check the real process duration time (in order to calculate the MRR values), to analyze the axial movement during the different stages of the machining process and, in general, to analyze and evaluate the proper functioning of the process. Assessing the unnecessary movements and retractions of the electrode helped to determine the quality of the consistency of the process. All the tests carried out in this research work were repeated twice, and the average values were used to show the results and to obtain the conclusions.

In order to evaluate the electrode wear, the loss in length and mass were measured. The length of the electrodes was measured before and after each EDM test to obtain the value of the longitudinal wear. In this paper, the length of the electrodes after finishing the corresponding EDM test is called the length of worn electrodes. These measurements were carried out in the EDM machine before taking out the electrodes from it, with an accuracy of micrometers. Moreover, the graphite electrodes were weighted before and after the tests. The weighting process was realized with a resolution of 0.1 mg in a Mettler Toledo scale (Model MS204S/01). It is known that graphite is a porous material, and as it was used submerged in a dielectric liquid, it was necessary to dry the electrodes thoroughly and to remove all dielectric liquid. To remove the ONA oil, each electrode was immersed in ether petroleum for 24 hours to extract the oil from the graphite’s pores. Afterwards, the electrodes were removed from the ether and placed in an oven at 100 °C until a constant weight was obtained. In this way, it was ensured that the real weight of the dried electrode was measured.

**Figure 5:**
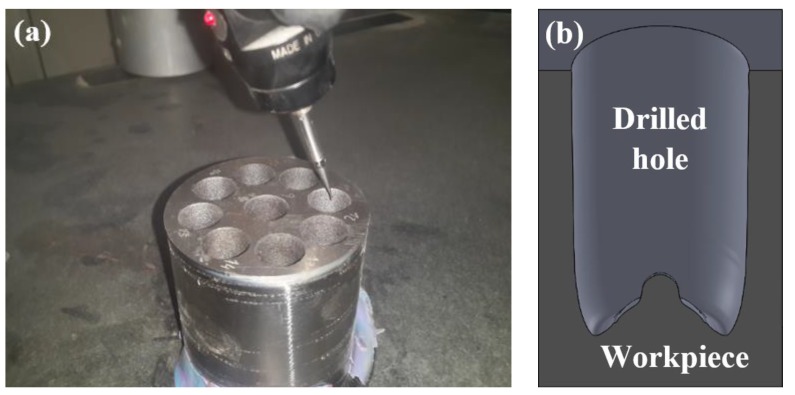
(**a**) The characterization of the machined cavities using Aberlink Coordinate Measuring Machine and (**b**) the 3-D geometry by Solidworks.

The generated cavities were measured using an Aberlink Coordinate Measuring Machine (CMM, Aberlink Innovative Metrology, Gloucestershire, UK) with a TP8 touch trigger manual indexing probe head and integrated probe, with the minimum available probe diameter of 1 mm, as shown in Figure 5a. The Aberlink 3-D measurement software was utilized to conduct the measurements of the vertical 2-D profile of the cavities. These 2-D profiles were fed into the Solidworks software to generate the 3-D geometry of the machined entities by revolving the captured profiles around their central axis, as shown in Figure 5b.

## 4. Results and Discussion

In this section, the results obtained in terms of TWR, hole taper angle and MRR will be analyzed. As discussed later, both hole taper angle and MRR will be affected by the tool wear, and thus, the latter will be discussed first.

### 4.1. Tool Wear Rate (TWR)

In all EDM machining research work, it is very important to evaluate the electrode wear because it directly affects the final machined shape obtained in the workpiece.

The tests carried out for this paper are not intended to replicate an industrial case study. In a real industrial process, several electrodes would be used in order to achieve the desired final shape in the workpiece. However, the purpose here was to assess which electrode cross section was the most suitable for self-flushing and, thus, would improve the machining results. With this in mind, only one electrode was used for each test. That is the reason why the electrode wear rate measured and evaluated in this paper may be more pronounced than in other papers or industrial processes where more than one electrode may be used.

The graphite electrodes were weighed before and after the tests, and the TWR was calculated as follows:

(1) $$\mathrm{TWR}=\;\frac{\mathrm{Electrode}’s\;\mathrm{initial}\;\mathrm{mass}\;(\mathrm{mg})\;-\;\mathrm{Electrode}’s\;\mathrm{final}\;\mathrm{mass}\;(\mathrm{mg})}{\mathrm{Machining}\;\mathrm{time}\;(s)}$$

As shown in Equation (1), the TWR evaluates the worn mass of the electrode per time unit. 

**Figure 6:**
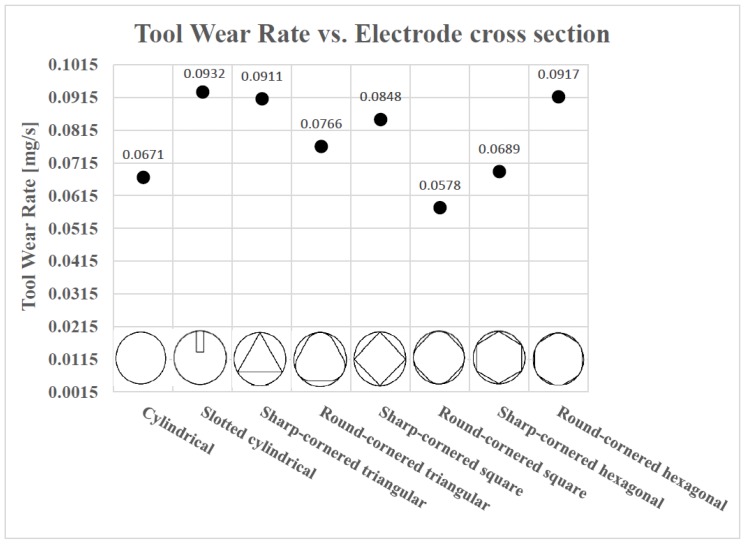
The Tool Wear Rate (TWR) for different shaped electrodes.

Figure 6 shows the TWR values of the different electrodes together with a picture of the initial cross section of each electrode.

**Figure 7:**
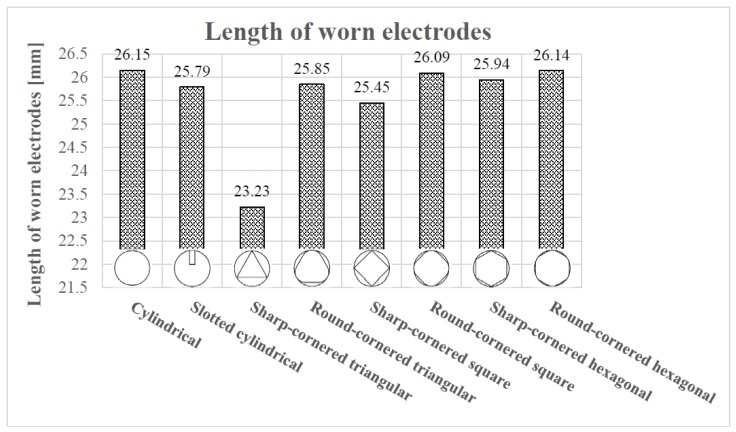
The length of worn electrodes for different shaped electrodes.

In Figure 6, it is not so difficult to observe that, generally, the TWR is lower in the case of shapes with rounded corners. Apart from that, in order to help obtain a wider perspective of this phenomenon, the length of worn electrodes after each EDM drilling test are collected in Table 5 and plotted in Figure 7.

From Table 5, it can be observed that the difference in the final length of the worn electrodes is not significant except in the case of the sharp-cornered triangle, especially since the mean of the worn electrodes’ length = 25.58 mm and standard deviation = 0.977 mm and the length of sharp-cornered triangular electrode is the only electrode that falls outside: mean ± two standard deviations. However, a more insightful conclusion can be drawn looking at the percentage of the electrode wear (electrode wear/original length of the electrode × 100). In particular, given that the original length of the electrodes was 30 mm, the electrode wears are found to vary between 3.85 mm (for the plain cylindrical electrode) and 6.77 mm (for the sharp corner-triangular electrode), equivalent to 12.83% and 22.57%, respectively.

As shown in Equation (1), the TWR was calculated from the initial and the final masses of the electrodes. If there is no significant difference in the final length of the worn electrodes, then it can be assumed that the difference in the increase of the electrode wear can be explained by the difference in volume of the electrode due to different electrode shapes used in the EDM trials. 

It has been mentioned that the TWR is lower in the case of round-cornered shapes. The unique exception among the non-cylindrical electrodes is the case of the round-cornered hexagonal electrode, which has a bigger TWR with respect to that of the sharp-cornered hexagonal electrode. However, the length of the worn round-cornered one is longer than the final length of the worn sharp-cornered hexagonal electrode, as it can be seen in Table 5.

Therefore, in order to explain why the round-cornered hexagonal electrode results in a more pronounced TWR than the sharp-cornered hexagonal electrode, it is necessary to focus on the narrowness of the debris egress gap. Because of the cross section of the electrodes, the debris egress gap forces the electrode to induce self-flushing during machining. However, if the aforementioned debris egress gap is not big enough to make a correct flushing of the dielectric liquid (observe, in Figure 4, the lowest values of the debris egress gap) possible, the wear in the lateral part of the electrodes will tend to increase. This effect will be more pronounced if the debris egress gap is divided into six, which is the case for the round-cornered hexagonal electrode. This is the reason why the TWR is bigger in the case of the round-cornered hexagonal electrode than in the sharp-cornered hexagonal. A similar explanation can be inferred for the case of the slotted cylindrical electrode. However, in this case, the difference in length compared with the cylindrical electrode is considerable. Thus, the high TWR value of the slotted cylindrical electrode is attributed to a combination of lateral and axial wear.

As for the relationship between the TWR and the debris egress gap of the electrode geometry, it is interesting to compare the results presented in Figure 6 with that of Figure 4. Comparing the results presented in both figures, it can be concluded that there is no linear relationship between the TWR and the debris egress gap of the electrode geometry. Nevertheless, if the aforementioned exceptions (round-cornered hexagonal, cylindrical with slot and cylindrical electrodes) are excluded, it can be observed that the TWR increases as the debris egress gap gets larger.

The larger the debris egress gap, the smaller the area of the cross section of the electrode is, making the electrode weaker and, thus, more susceptible to wear. A bigger debris egress gap results in a thinner and weaker electrode when the machining parameters are the same for all the electrodes. Hence, in the case of the electrodes with a bigger debris egress gap, the discharge energy is more concentrated as the contact area between the electrode and the workpiece is smaller. This results in a locally more aggressive erosion process and a higher TWR. 

**Figure 8:**
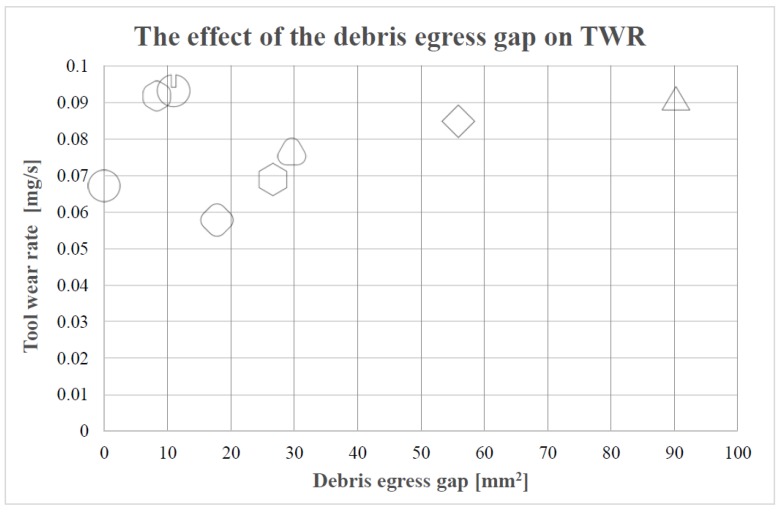
The relationship between TWR and the debris egress gap.

For a better understanding of the results, the resultant TWR vs. debris egress gap for different electrodes are plotted in Figure 8.

In particular, with the reduction in the debris egress gap, TWR tends to decrease. Thus, since the debris egress gap in the case of the cross sections of rounded corners are smaller than those related to the sharp cornered electrodes, it is possible to understand why, in general, the TWR in the case of round-cornered electrodes is lower than in the case of the sharp-cornered ones. 

Finally, the case of the cylindrical electrode is different from the other cases, as no self-flushing is achieved with this geometry. This is the reason why the lateral wear is not pronounced in this case. These results confirmed the initial hypothesis that shaped electrodes could exhibit a higher TWR. Nonetheless, the TWR value of the cylindrical electrode is not the lowest value among all the different electrodes, as it was expected at the beginning. The round-cornered square electrode presents a lower TWR value. Further research work will be necessary in order to clarify this result. 

To sum up, it can be concluded that, generally, TWR is lower if the debris egress gap is smaller, provided that the debris egress gap is not too small or narrow for self-pumping. Otherwise, the TWR will be increased due to the lateral electrode wear. Moreover, in general, round-cornered electrodes offer lower TWR values than sharp-cornered electrodes. Finally, the cylindrical electrode shows a low TWR but not the lowest.

### 4.2. Hole Taper Angle

The hole taper is a very common dimensional defect that occurs in cutting and drilling operations. It is characterized by obtaining a wider diameter at the top than that at the bottom in the case of drilling and getting a larger width at the entry than that at the exit in cutting.

**Figure 9:**
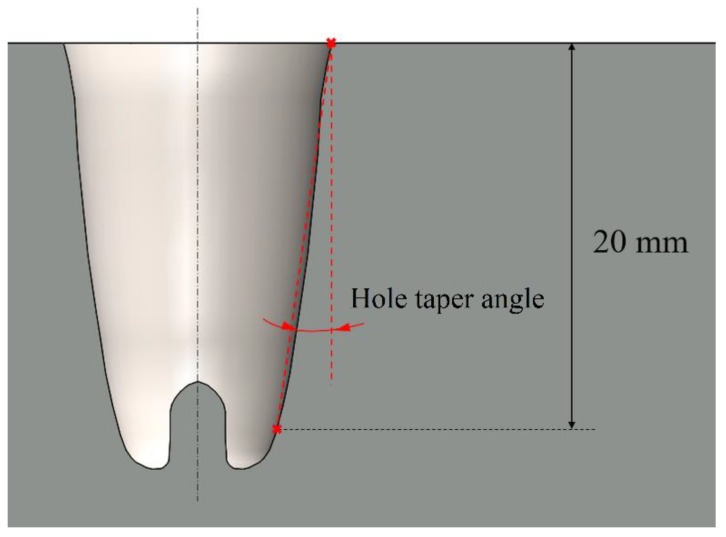
The geometrical definition of the hole taper angle.

The hole taper usually is an undesirable characteristic, and it is typically evaluated using the hole taper angle. In this work, the inner diameter was measured at a depth of 20 mm, as it was neither possible nor reliable to determine the diameter at the bottom of the hole. A schematic definition of the hole taper angle is shown in Figure 9.

The main reason for the appearance of the mentioned hole taper during the EDM drilling process is the wear of the electrode. Many different researchers have investigated minimizing electrode wear and, thus, the hole taper angle. During the EDM drilling of difficult-to-cut materials, especially in micromachining, preventing electrode wear means enhancing productivity [37]. 

**Figure 10:**
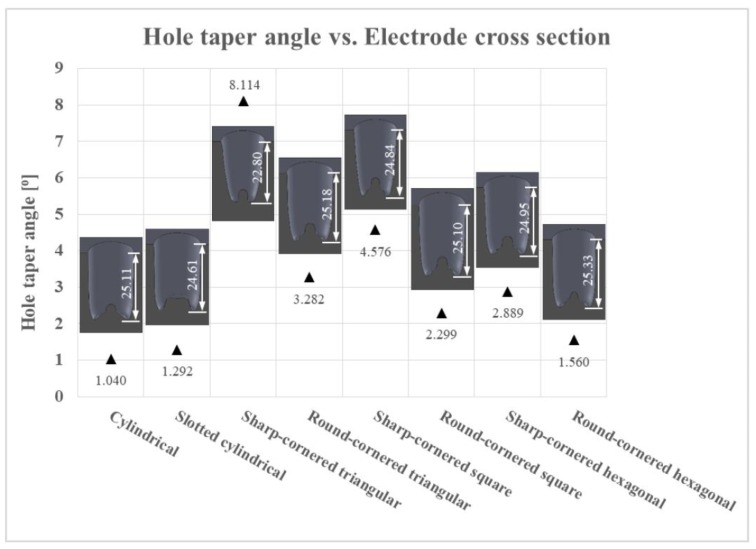
The hole taper angle values in relation with the different electrode cross sections (the dimensions are in mm).

Figure 10 shows the angle of the hole taper and the actual depth of the machined holes in millimeters. The values measured in the holes machined with the eight different electrode geometries varied from 1.040 to 8.110. There was a significant variation of the hole taper angle depending on the electrode geometry.

It is not so difficult to see that, for all drilled holes, no flat bottoms were obtained but that bottom surfaces are uneven with defects (large cone) in the center. These defects are mainly attributed to an increased current density at the bottom corner of the electrode, which results in the removal of more material at the edges than the removal from the central portion of the electrode, which in turn produces a large cone in the center of the bottom surface of the electrodes, as seen in Figure 10 [38]. 

**Figure 11:**
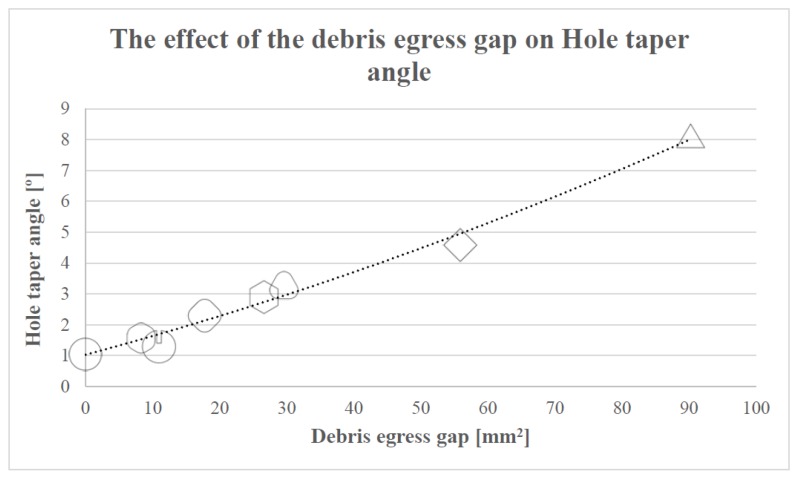
The relationship between the hole taper angle and the debris egress gap.

Comparing the results presented in Figure 10 with the percentages of debris egress gap of different electrodes represented in Figure 4, it can be concluded that the larger the debris egress gap was, the larger the hole taper angle would be. The electrode is more likely to wear, associated with a larger hole taper angle, if its cross-sectional area is smaller (resulting in a larger debris egress gap). This statement can be clearly observed in Figure 11.

In short, the best result with regards to the hole taper angle was obtained with the cylindrical electrode, which confirmed the initial hypothesis about the hole taper angle. The cylindrical electrode was followed by the slotted cylindrical and the round-cornered hexagonal electrodes, as they present the lowest values of the hole taper angle. In contrast, the sharp-cornered triangular electrode shows the worst results. This is due to the weakness of the electrode geometry and, thus, its tendency to wear axially. Sharp-cornered square and round-cornered triangular electrodes also produced relatively poor results.

### 4.3. Material Removal Rate (MRR)

The material removal rate (g/min) is used in this research as a criterion of machinability to assess the performance of the EDM process for different electrode geometries. In particular, the volumes of the 3-D geometry of the machined cavities (holes) were quantified using SolidWorks, enabling the determination of the removed mass of each hole by utilizing the density of Ti6Al4V. Please note that, although the procedure followed to quantify the removed material is an indirect method, it prevents the workpiece from being taken off for frequent assessment after each EDM trial, sustaining unchanged workpiece setting conditions and minimizing the associated uncertainty errors. Moreover, these indirect quantifications are based on direct volume measurements of the machined cavities as explained in Section 3.3 and shown in Figure 5a.

To determine the MRR for each EDM machining trial, the amount of removed material (in grams) was divided by the corresponding machining time and recorded using the data acquisition system previously explained in Section 3.3. Therefore, the MRR was calculated as follows:

(2) $$\mathrm{MRR}=\;\frac{\mathrm{Volume}\;\mathrm{of}\;\mathrm{machined}\;\mathrm{hole}\;\left[{\mathrm{mm}}^{3}\right]\;\times \;{\mathrm{Workpiece}\;\mathrm{density}\;[g/\mathrm{mm}}^{3}]}{\mathrm{Machining}\;\mathrm{time}\;[\min]}$$

**Figure 12:**
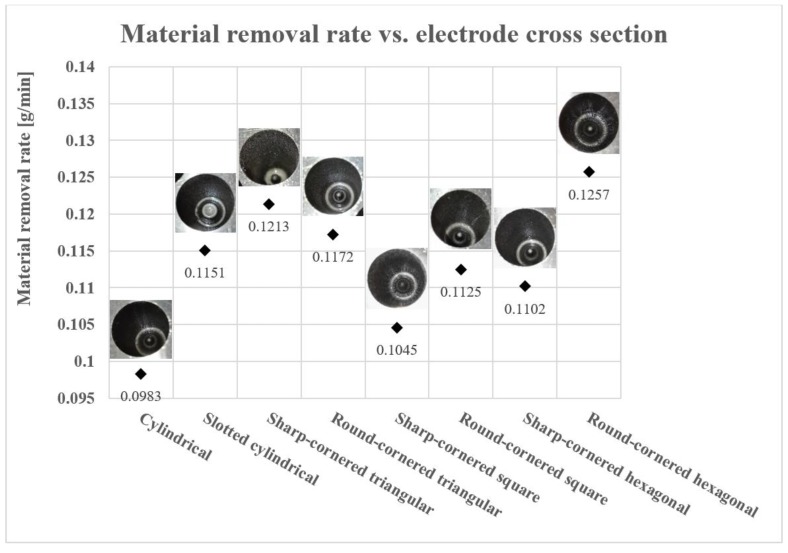
The Material Removal Rate (MRR) for different shaped electrodes.

Figure 12 shows the resultant MRR for the eight different electrode geometries. In these EDM trials, as previously explained, a shaped electrode simultaneously rotates and retracts, which promotes self-pumping of the dielectric fluid [26,31]. This evacuates debris along the helical paths generated between the moving shaped electrode and the machined hole (see Figure 3b) and, thus, induces self-flushing of the debris through the gap at the top surface. This assumption is clearly confirmed when looking at the results, as the minimum MRR value (0.0983 m/min) was obtained for the solid cylindrical electrode, with no debris egress gap. The results show that the slotted cylindrical electrode gave a better result (MRR = 0.1151 g/min), which can be attributed to the debris egress induced through the tailor-made slot [32].

It is noticeable that, apart from the triangular shape, the cross sections with rounded corners produced higher MRRs than those obtained with sharp-corners. This can be ascribed to the larger cross-sectional areas of the square and hexagonal round-cornered cross sections, associated with larger machining contact areas than those with sharp-corners. The exception was the triangular electrode, where the sharp-cornered electrode exhibited a higher MRR than the round-cornered. However, in the case of the sharp-cornered triangular electrodes, the depth of the machined hole was less (22.80 mm) than the other machined holes, which had depths between 24.61 mm and 25.33 mm (see Table 5). It is worth stating that the MRR decreases with the depth of machining since debris evacuation becomes increasingly difficult the deeper the hole. This means the high MRR in the case of the sharp-cornered triangular electrode was due to the large amount of material removed in a short time at a relatively short depth, as this particular trial was associated with a high TWR and a large hole taper angle (see Figure 6 and Figure 10). Hence, it is possible to conclude that it is not fair to compare the MRR value obtained with the sharp-cornered triangular electrode with the MRR values obtained in the other tests because the latter were obtained for deeper holes. 

When comparing the performance of different electrode shapes, the maximum MRR value (0.1257 g/min) was achieved with the round-cornered hexagonal electrode. This can be explained by the large contact area between the electrode and the processed material that increased the rate of erosion of the material. In addition, the high value of MRR obtained in the case of a round-cornered hexagonal electrode can be attributed to the self-pumping of the dielectric fluid, in particular, because this self-pumping action is uniformly distributed into six different regions along the circumference of the drilled hole. This homogenous flushing (debris egress) leads to a more consistent machining process and, eventually, a higher MRR.

**Figure 13:**
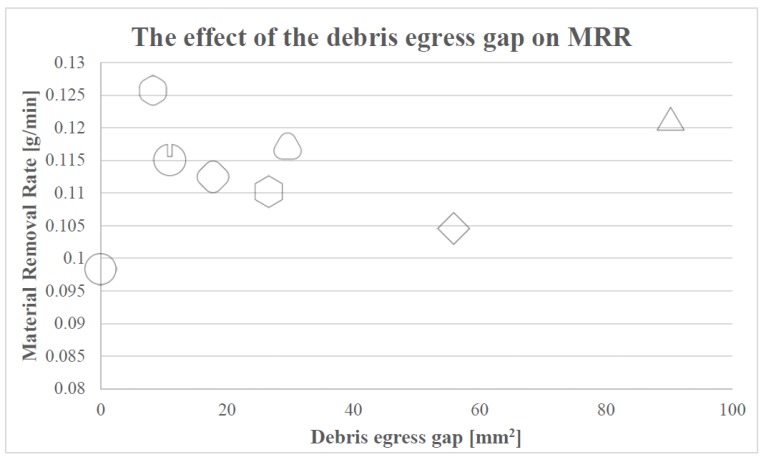
The relationship between the MRR and the debris egress gap.

In Figure 13, it can be observed that, in general, the bigger the contact area between the electrode and the processed material is and, thus, the smaller the debris egress gap is, the higher the MRR obtained is during these EDM trials. The observed relationship between the achievable MRR and debris egress gap has two exemptions to its general tendency, namely the results obtained in case of the sharp-cornered triangular electrode and the cylindrical electrode, and the possible reasons for this were formerly discussed. 

To sum up, one can say that the initial hypothesis for the MRR was proved. Especially, the cylindrical electrode obtained the lowest MRR value when compared with the performance of shaped electrodes. Nevertheless, it is worth emphasizing that, in general, a higher MRR was achieved when round-cornered shaped electrodes were utilized. 

**Figure 14:**
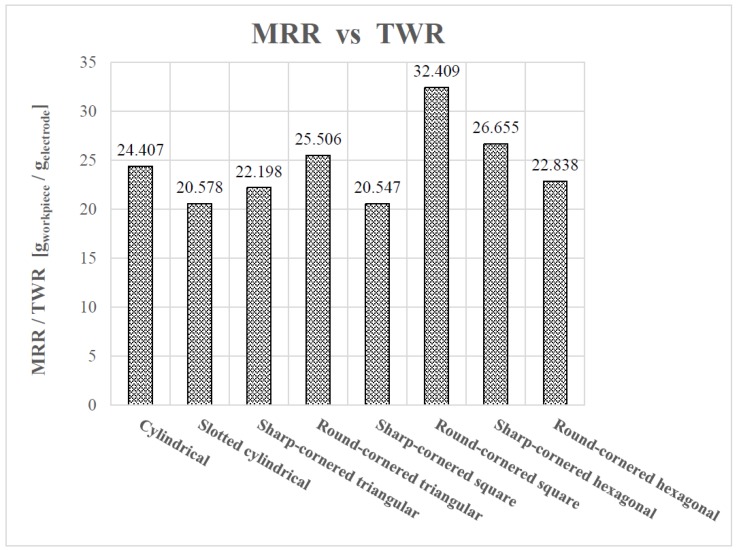
The ratio of the MRR to the TWR.

To provide a more comprehensive overview of the electrode’s performance, the ratio of the MRR to the TWR for each electrode is presented in Figure 14.

From Figure 14, it can be determined that the round-cornered square electrode provides the more preferable performance, balancing the MRR and TWR. Moreover, the sharp-cornered hexagonal electrode shows a better performance when compared with the remaining electrodes.

Finally, it is worth emphasizing that the election of the electrode shape should depend on the process criterion that the user would like to optimize, which does not necessarily have to be an equilibrium between the different attributes.

## 5. Conclusions

This paper has presented an experimental study of self-flushing in the EDM drilling of Ti6Al4V using rotating shaped electrodes. In particular, EDM tests to drill blind holes were carried out using rotating graphite electrodes with eight different cross sections, where TWR, hole taper angle and MRR were used as quality marks.

The main conclusions drawn are as follows:

- The investigation revealed a high correlation between the electrode geometry and feasibility of self-flushing when other EDM parameters, such as rotational speed, workpiece material and electrode material, were kept constant. In particular, the results show a greater capacity of the self-flushing technique of the shaped electrodes when compared with the simple cylindrical electrode.
- In general, the results demonstrated that round-corner shaped electrodes are associated with a lower TWR and hole taper angle and a higher MRR when compared to electrodes with sharp corners. In particular, the highest and lowest TWR values (0.0932 mg/s and 0.0578 mg/s, respectively) were found in the case of the slotted cylindrical electrode and the round-cornered square electrode, respectively. Although, the electrode wear varied between 3.85 mm and 7.2 mm, the percentage of the electrode wear was found quite significant and ranged between 12.83% and 22.57%.
- Moreover, the largest hole taper angle was produced for the sharp-cornered triangular electrode (8.114°), and the smallest value (1.040°) was achieved with the cylindrical electrode. However, low hole taper angles (1.292°) were also obtained with the slotted cylindrical and round-cornered hexagonal electrodes. Furthermore, the investigation has shown that the round-cornered hexagonal electrode exhibited the maximum MRR (0.1257 g/min), while the cylindrical electrode gave the minimum MRR (0.0983 g/min).
- The findings of this research study show diverse trends for the effect of electrodes shapes on the EDM process aspects, namely TWR, hole taper angle and MRR. For a high MRR, the round-cornered hexagonal electrode is the first choice, though a cylindrical electrode has to be used if a low hole taper is to be attained, while a low TWR can be attained using a round-cornered square electrode.
- The results of this research confirmed the initial hypothesis that, in general, using shaped electrodes gave better results in terms of MRR but not the case of TWR and hole taper angle when compared with the performance of the plain cylindrical electrode. Moreover, the more robust the geometry of the electrode was, the lower the value of the TWR and hole taper angle could be achieved. Nevertheless, if the debris egress gap is too narrow, the TWR will increase due to the difference in the volume of the electrode that depends on the electrode shape used.

In future work, these possible research lines are detected:

- In future work, a further investigation of self-flushing EDM processes should be conducted utilizing a wider range of electrodes with different cross sections, different radii of the rounded-corners and other polygonal electrodes, such as pentagonal or rectangular. Moreover, the self-flushing phenomenon should be evaluated in different electrode materials, for instance in copper, copper tungsten or brass. Furthermore, combinations of different tool cross sections and other flushing approaches (e.g., ultrasonic-assisted technique) should be considered. Moreover, the effect of different speeds of rotation can also be examined. In addition, the value of the debris egress gap and its distribution over the cross section could be optimized using fluid-dynamics simulation.
- It is important to emphasize that the highest MRR and the lowest tool wear were not achieved using a single electrode, and thus, further research work could be conducted along these lines to improve the machining efficiency in EDM machining of blind holes. This also could entail examining new EDM strategies (the number of electrodes and different electrode shapes) to machine deep holes with a high MRR, a low TWR and a high dimensional accuracy.
- Finally, an important research work should be conducted with regards to the influence of the usage of the rotating shaped electrodes and the self-flushing techniques on the surface quality and integrity of the workpiece.

## Figures and Tables

**Table 1:** The chemical composition of Ti6Al4V titanium alloy (weight %).

Ti	Al	V	Fe	Others
89.2	6.1	4.1	0.2	Balance

**Table 2:** The physical properties of Ti6Al4V titanium alloy.

**Density, ρ (g/cm^3^)**	4.43
**Melting Temperature, T_m_ (°C)**	1660
**Hardness, HRc**	36
**Thermal Conductivity, κ (W/mK)**	6.7
**Specific Heat Capacity, C_p_ (J/kg °C)**	526.3

**Table 3:** The discharge conditions.

**Mean discharge Current, I (A)**	32
**Ionisation Voltage, U (V)**	80
**Pulse-On Time, t_i_ (µs)**	100
**Pulse-Off Time, t_o_ (µs)**	200
**Tool Polarity**	Positive
**Dielectric**	ONA oil
**Rotational Speed (rpm)**	40

**Table 4:** The electrode designation, 3-D representation and cross section.

Electrode Designation	3-D Geometry and Cross Section
Cylindrical electrode	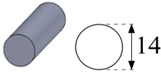
Slotted cylindrical electrode	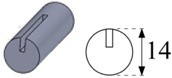
Sharp-cornered triangular electrode	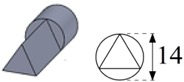
Round-cornered triangular electrode	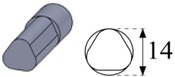
Sharp-cornered square electrode	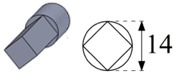
Round-cornered square electrode	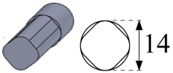
Sharp-cornered hexagonal electrode	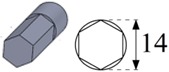
Round-cornered hexagonal electrode	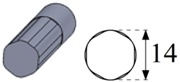

**Table 5:** The length of worn electrodes (the original length of all electrodes was 30 mm).

Shape	Length of Worn Electrodes (mm)
Cylindrical	26.15
Slotted cylindrical	25.79
Sharp-cornered triangular	23.23
Round-cornered triangular	25.85
Sharp-cornered square	25.45
Round-cornered square	26.09
Sharp-cornered hexagonal	25.94
Round-cornered hexagonal	26.14
